# How big is the ‘next big thing’? Estimating the burden of non–communicable diseases in low– and middle–income countries

**DOI:** 10.7189/jogh.02.020101

**Published:** 2012-12

**Authors:** Kit Yee Chan, Davies Adeloye, Liz Grant, Ivana Kolčić, Ana Marušić

**Affiliations:** 1 Nossal Institute for Global Health, University of Melbourne, Melbourne, Australia; 2 Department of Health Policy and Management, School of Public Health, Peking University Health Science Centre, Beijing, China; 3 Centre for Population Health Sciences and Global Health Academy, The University of Edinburgh Medical School, Edinburgh, Scotland, UK; 4 Croatian Centre for Global Health, University of Split School of Medicine, Split, Croatia; 5 Department of Research in Biomedicine and Health, University of Split School of Medicine, Split, Croatia

Non-communicable causes of death and disability will dominate global health agenda for the foreseeable future. The progress in addressing their burden and achieving measurable reduction in low– and middle–income countries (LMICs) will likely require similar steps that were effective in reducing maternal and child mortality globally: (i) defining the size of the burden and the main causes responsible for the majority of the burden; (ii) understanding the most important risk factors and their importance in different contexts; (iii) systematically assessing the effectiveness and cost of the interventions that are feasible and available in LMICs; and (iv) formulating evidence–based health policies that will define appropriate health care and health research priorities to tackle the burden in the most cost–effective way.

Over the past year the pandemic of non–communicable diseases (NCDs) has become a key focus of global political agenda. At the United Nations' high–level meeting on the prevention and control of NCDs in September 2011, a general consensus has been reached that NCDs were already the leading causes of death in all world regions and that their burden is increasing rapidly [1]. The rate of this increase is particularly striking in low– and middle–income countries (LMICs), where life expectancy is increasing as a result of improved socio–economic conditions [2]. It is expected that by the year 2030, NCDs could become responsible for 52 million deaths [3]. In LMICs, health systems will face considerable challenge in adjusting to the rapidly growing demand for services, and this could in turn become an additional significant barrier to achieving the Millennium Development Goals [2]. As a result, many parallel advocacy efforts for tackling NCDs are taking place, with a particular focus on heart disease, cancer, respiratory diseases, diabetes and stroke [4]. A number of interventions have been outlined that could have immediate preventive effect and slow down the pandemic, such as tobacco control, improved diet, exercise and decreased alcohol intake [4].

The release of the new global burden of disease (GBD) estimates for the year 2010, by the Institute for Health Metrics and Evaluation (IHME) at the University of Washington in Seattle, is anticipated with great interest [5]. The new revision is expected to show substantial progress in the reduction of maternal and child mortality in the LMICs over the past two decades. However, many fear that there will be hardly any measurable progress in improving health and survival of adult populations in LMICs. The UN conference in 2011 and the publication of the new GBD estimates could therefore mark the beginning of the era in which non–communicable causes of death and disability will dominate global health agenda for the foreseeable future. The progress in addressing their burden and achieving measurable reduction in LMICs will likely require similar steps that were effective in reducing maternal and child mortality globally: (i) defining the size of the burden and the main causes responsible for the majority of this burden; (ii) understanding the most important risk factors and their importance in different contexts; (iii) systematically assessing the effectiveness and cost of the interventions that are feasible and available within the contexts of different LMICs; and (iv) formulating evidence–based health policies that will define appropriate health care and health research priorities to tackle the burden in the most cost–effective way.

The first step in this process is to measure the burden of NCDs in LMICs. This is a challenging task given the scarcity of available data, inconsistency in case definitions of the measured diseases, differences in reporting of results (eg, age groups) used by different investigators, lack of funding, research infrastructure and capacity for community–based studies in LMICs, changing definitions of diseases over time, low transcultural adaptability of screening instruments, and many others [6-8]. Methodological approaches that could take into account the diversity and scarcity in the available information and produce acceptable regional estimates using transparent and sound methodological approaches are urgently needed. Furthermore, the international research community could benefit from clear guidelines on conducting epidemiological studies in LMICs that could inform burden of disease analyses, so that research results are comparable and leading to more reliable estimates.

In the current issue of the *Journal of Global Health*, we are publishing several studies that attempt to summarise information on the burden of non–communicable diseases and provide estimates for a region that has traditionally been considered “information gaps”: the African continent. The papers by George-Carey et al. [9], Paul et al. [10], Graham et al. [11] and Dowman et al. [12] provide the first systematic estimates of the burden of dementia, epilepsy and rheumatoid arthritis, respectively. In addition, Reidpath and Allotey provide an authoritative viewpoint on the changing chronic disease management in LMICs [13], Moten et al. discuss the challenge of equitable building of public health infrastructure in low resource settings [14], Kolcic warns of the “double burden of malnutrition” as a silent driver of the NCD pandemic [15], while Maher and Sridhar address the role of political priority in the global fight against NCDs [16]. In the future issues of our journal, we will increasingly welcome similar attempts to quantify disease burden, the role of risk factors and the effectiveness of interventions targeted at reducing NCDs in low resource settings.
